# Prediction of total knee replacement using deep learning analysis of knee MRI

**DOI:** 10.1038/s41598-023-33934-1

**Published:** 2023-04-28

**Authors:** Haresh Rengaraj Rajamohan, Tianyu Wang, Kevin Leung, Gregory Chang, Kyunghyun Cho, Richard Kijowski, Cem M. Deniz

**Affiliations:** 1 Center for Data Science, New York University, 60 5th Ave, New York, NY 10011 USA; 2 Courant Institute of Mathematical Sciences, New York University, 251 Mercer St, New York, NY 10012 USA; 3 Department of Radiology, New York University Langone Health, 660 1st Ave, New York, NY 10016 USA; 4 Bernard and Irene Schwartz Center for Biomedical Imaging, New York University Langone Health, 650 First Avenue, Room 418, New York, NY 10016 USA

**Keywords:** Prognostic markers, Outcomes research, Medical imaging, Prognosis, Risk factors

## Abstract

Current methods for assessing knee osteoarthritis (OA) do not provide comprehensive information to make robust and accurate outcome predictions. Deep learning (DL) risk assessment models were developed to predict the progression of knee OA to total knee replacement (TKR) over a 108-month follow-up period using baseline knee MRI. Participants of our retrospective study consisted of 353 case–control pairs of subjects from the Osteoarthritis Initiative with and without TKR over a 108-month follow-up period matched according to age, sex, ethnicity, and body mass index. A traditional risk assessment model was created to predict TKR using baseline clinical risk factors. DL models were created to predict TKR using baseline knee radiographs and MRI. All DL models had significantly higher (*p* < 0.001) AUCs than the traditional model. The MRI and radiograph ensemble model and MRI ensemble model (where TKR risk predicted by several contrast-specific DL models were averaged to get the ensemble TKR risk prediction) had the highest AUCs of 0.90 (80% sensitivity and 85% specificity) and 0.89 (79% sensitivity and 86% specificity), respectively, which were significantly higher (*p* < 0.05) than the AUCs of the radiograph and multiple MRI models (where the DL models were trained to predict TKR risk using single contrast or 2 contrasts together as input). DL models using baseline MRI had a higher diagnostic performance for predicting TKR than a traditional model using baseline clinical risk factors and a DL model using baseline knee radiographs.

## Introduction

Knee osteoarthritis (OA) is one of the most prevalent and disabling chronic diseases, occurring in 10% of men and 13% of women over 60 years of age^1^ and contributing to more than $27 billion in annual healthcare expenditures in the Unites States alone^2^. Conservative treatment options including weight loss, aerobic activity, and muscle strengthening exercises can alleviate symptoms and potentially slow the rate of disease progression in patients with knee OA^3,4^, but are most effective when initiated during the early stages of the disease^5,6^. Thus, a preventive strategy that targets patients without advanced knee joint degeneration at high risk for OA progression could optimize the effectiveness of current conservative interventions. In addition, identifying individuals with knee OA at high risk for disease progression would help select the most optimal subjects for inclusion in clinical OA drug trials. This would facilitate the conduct of more affordable and shorter-duration studies involving smaller number of subjects that would expedite the development of new disease modifying OA therapies^5^.

Magnetic resonance imaging (MRI) is a highly useful imaging modality for evaluating knee OA as it can assess all joint structures including cartilage, bone, meniscus, ligament, and synovium that can be sources of pain in patients with the disease^7^. Semi-quantitative measures of structural features of knee joint degeneration on MRI have been shown to be associated with knee OA progression including an increased risk for future total knee replacement (TKR)^8–13^. However, obtaining semi-quantitative parameters requires a reader to assess each individual structural feature in each region of the knee joint using categorical based scoring systems. This is a time-consuming and reader-dependent process, which would be difficult to incorporate into widespread, cost-effective OA risk assessment models. Thus, more efficient and reliable methods are needed to extract useful prognostic information from imaging studies.

Deep learning (DL) is an advanced form of artificial intelligence that has been successfully used for various medical imaging applications. Especially notable is a subclass of DL algorithms termed convolutional neural networks (CNNs), which have dominated the field of computer vision and surpassed human abilities in many important tasks^14^. Given enough training data, a CNN could automatically learn a representative subset of features such as structural features of knee joint degeneration on MRI associated with knee OA progression. DL offers an exciting new opportunity for rapid, fully automated extraction of useful prognostic information from imaging studies that could potentially be used to assess the risk of OA progression in every patient being evaluated in clinical practice with knee radiographs and MRI. Our study was performed to develop DL risk assessment models to predict the progression of knee OA to TKR over a 108-month follow-up period using baseline knee MRI. We hypothesize that DL models using baseline MRI would have higher diagnostic performance for predicting TKR than a traditional model using baseline clinical risk factors and a DL model using baseline knee radiographs.

## Methods

### Subject cohort

Our retrospective study was performed using knees selected from subjects in the publicly available Osteoarthritis Initiative (OAI) and Multicenter Osteoarthritis Study (MOST) databases. The OAI database contains demographic and clinical information, radiographs, and MRI examinations from 4796 subjects between 45 and 79 years of age with or at risk for knee OA evaluated at baseline and 12, 24, 36, 48, 60, 72, 84, and 108-month follow-up^15^. The MOST database contains the same information and imaging studies from 3026 subjects between 50 and 79 years of age with or at risk for knee OA evaluated at baseline and 15, 30, 60, 84, 144, and 168-month follow-up. The OAI and MOST were approved by the Internal Review Boards at University of California at San Francisco, Boston University Medical Center, and each individual clinical recruitment site and was performed in compliance with the Declaration of Helsinki. All subjects signed written informed consent.

### Training and validation group

A training and validation group from the OAI database was selected to train the models that are not biased towards age, BMI, sex, and race, and use only features on radiographs and MRI to predict TKR. A balanced case–control cohort was selected by matching case subjects and control subjects using baseline demographic variables associated with knee OA progression including age, sex, ethnicity, and body mass index (BMI). Case subjects were defined as individuals who underwent a TKR in either knee after the baseline enrollment date, while control subjects were defined as individuals who appeared at the 108-month follow-up visit and had not undergone a TKR in either knee. If a patient underwent TKR in both knees during OAI data collection, the knee that first underwent TKR was included. Each case patient with TKR was matched to a control subject without TKR who was the same age, sex, and ethnicity and with an additional constraint on the baseline BMI within a 10% tolerance. The data set from case–control pairs contained either the left or right knee from each case and control subject.

**Figure 1:**
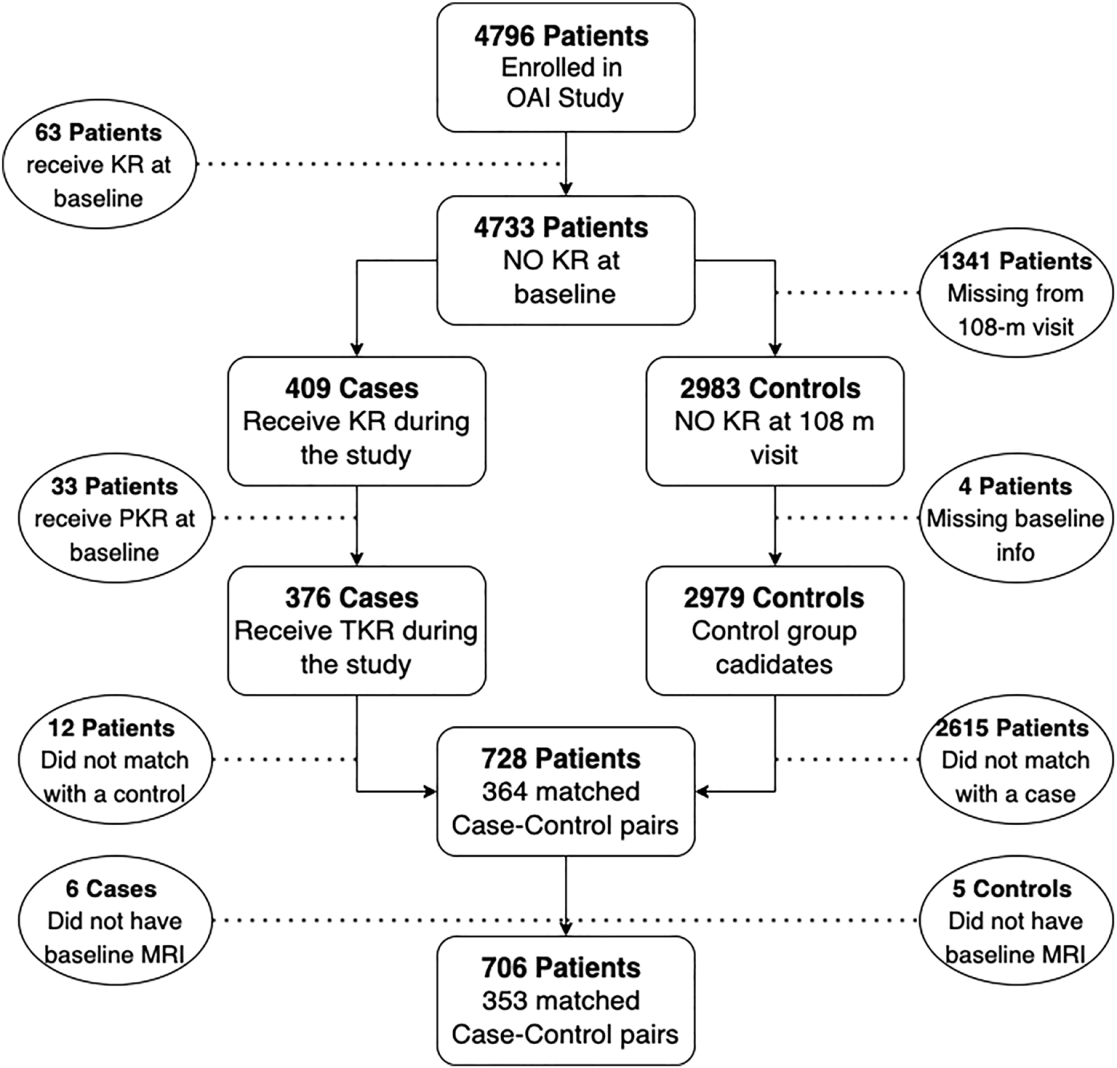
Selection of the 353 case–control pairs of subjects in the OAI database with and without TKR over a 108-month follow-up period that comprised the subject cohort.

A total of 353 case–control pairs were identified from the 4796 subjects in the OAI database. Subjects were excluded if they had TKR at baseline, received partial knee replacement over the course of follow-up, were missing baseline or 108-month follow-up information, or did not match with a case or control subject. A summary of the selection of case–control pairs is illustrated in Fig. 1. Study cohort characteristics are summarized in Supplementary Table 1. All 706 matched subjects had baseline standing posterior-anterior knee radiographs, knee MRI examinations, and clinical outcome measures including the Western Ontario and McMaster Universities Osteoarthritis Index (WOMAC)^16^ and Quality of Life from Knee Injury and Osteoarthritis Outcome Score (KOOS QoL)^17^. A subset of 270 subjects had baseline semi-quantitative MRI scores for the severity of cartilage loss and bone marrow edema lesions on 14 knee articular surfaces using the MRI Osteoarthritis Knee Score (MOAKS) system^18^ provided by central reading of the National Institute of Health OA Biomarkers Consortium Project.

### Total knee replacement risk assessment models

**Figure 2:**
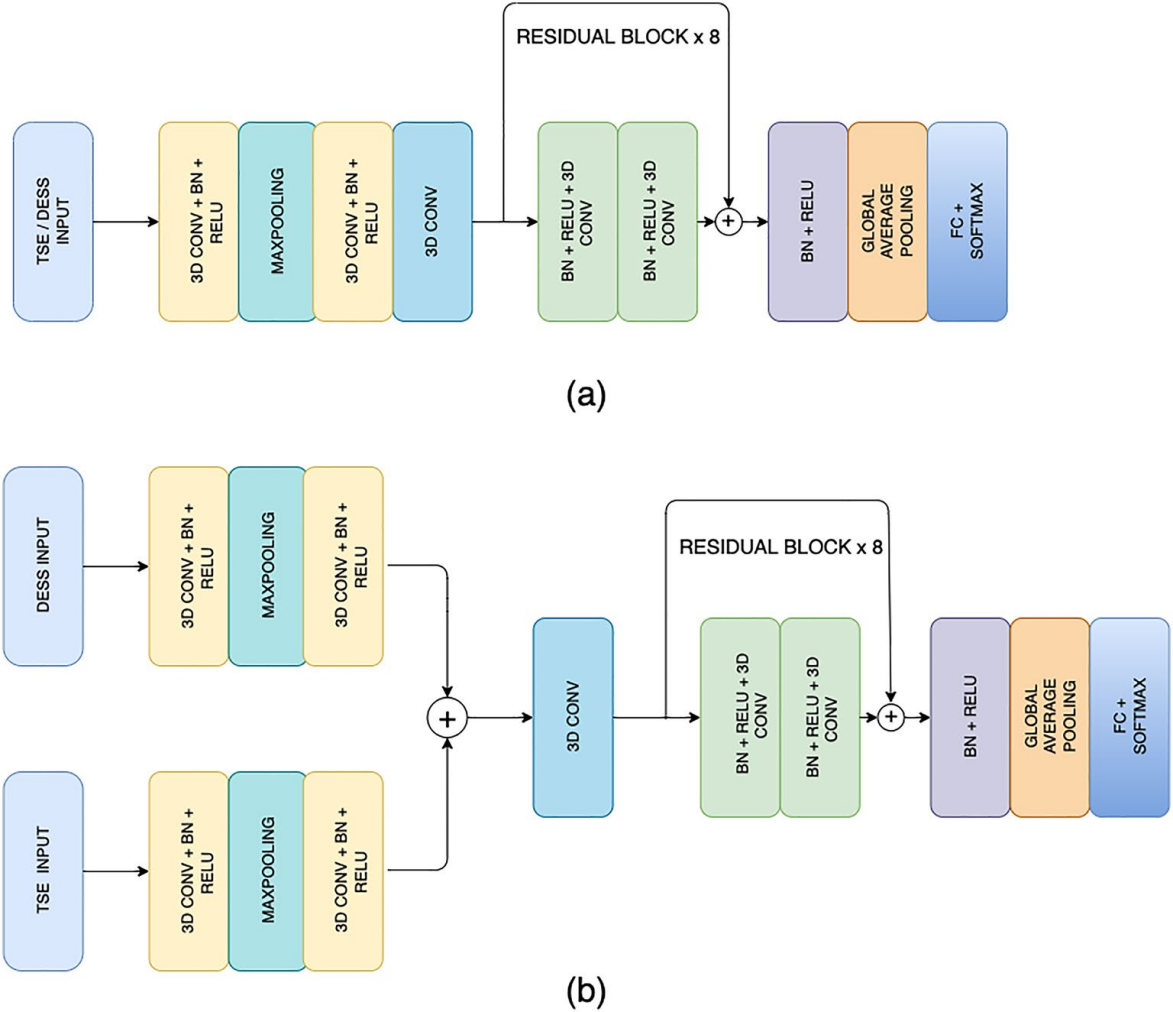
(**a**) Architecture of the coronal intermediate-weighted turbo spin-echo (IW-TSE), sagittal fat-suppressed intermediate-weighted turbo spin-echo (FS-IW-TSE), and fat-suppressed three-dimensional dual-echo in steady-state (DESS) models. (**b**) Architecture of the multi-input MRI model.

The MRI examinations for all subjects in the training and validation group included coronal intermediate-weighted turbo spin-echo (IW-TSE), sagittal fat-suppressed intermediate-weighted turbo spin-echo (FS-IW-TSE), and sagittal fat-suppressed three-dimensional (3D) dual-echo in steady state (DESS) sequences performed on a 3.0T whole-body scanner (Magnetom Trio, Siemens Healthcare, Erlangen, Germany) with the imaging parameters shown in Supplementary Table 2. To determine the best OA risk assessment model, multiple models were developed using analysis of each individual sequence and using two different approaches for combined analysis of multiple sequences. Three separate MRI models were created for the IW-TSE, FS-IW-TSE, and DESS images as each tissue contrast has advantages for evaluating different knee joint structures^19^. A multi-input MRI model was also developed by inherently combining information from FS-IW-TSE and DESS images in the same CNN architecture (Fig. 2). All MRI models were created with CNN architectures using conventional residual blocks with extension to 3D. The use of 3D CNN architectures allowed 3D analysis of the DESS sequence without the need to evaluate reformatted images in different planes. The outcome predictions for the models were a confidence value between 0 and 1 indicating the likelihood for TKR. Details regarding the CNN architectures used in the IW-TSE, FS-IW-TSE, DESS, and multi-input MRI models are included in the Supplementary Materials. The source code for this study is available at [Link provided after review].

A radiograph model for predicting TKR was created from a previous study using a CNN architecture adapted from ResNet with 34 layers, which analyzed baseline standing posterior-anterior knee radiographs and provided a confidence value between 0 and 1 as an outcome prediction indicating the likelihood for TKR^20^. The OAI cohort used in this work is a subset of the cohort from^20^ and during evaluation, it was ensured that there was no leakage between the training sets in^20^ and the test set in our work. An MRI ensemble model was created by averaging the outcome predictions of the IW-TSE, FS-IW-TSE, and DESS models. In comparison to the multi-input MRI model that analyzed the different MRI sequences together to predict TKR, the MRI ensemble model averaged the outcome predictions of the models analyzing the different sequences separately. An MRI and radiograph ensemble model was created by averaging the outcome predictions of the ensemble MRI and radiograph models.

A traditional risk assessment model for predicting TKR was created using multi-layer perceptrons, which analyzed baseline clinical risk factors including BMI, WOMAC and contralateral WOMAC, and KOOS QoL^21,22^. Input features were normalized to unit mean and zero variance using training dataset statistics. The multi-layer perceptrons passed the input features through 2 fully-connected hidden layers of size 256 with ReLU activation and batch normalization and provided a confidence value between 0 and 1 as an outcome prediction indicating the likelihood for TKR.

### Model training and validation

Model training and validation was performed using sevenfold nested cross-validation. The 353 case–control pairs were split equally into 7 parts, with the number of pairs used for model training ranging between 250 and 256 and the number of pairs used for model validation and testing ranging between 47 and 52. The dataset splits were performed randomly using a random data generator in Python (version 2.7, Python Software Foundation, Wilmington, DE). Details regarding model training, evaluation, and dataset splits are included in the Supplementary Materials.

### Model evaluation on internal and external testing groups

The models were evaluated on an internal hold-out testing group from the knees of the remaining 4090 subjects in the OAI database that were not involved in model training and validation and who were evaluated using the same MRI protocol and same MRI scanner. Among the remaining 4090 subjects, there were 32 case knees in 27 subjects that underwent TKR and 7891 control knees in 4034 subjects that did not undergo TKR between the baseline and 108-month follow-up periods. The remaining 257 knee in 216 subjects were excluded due to missing baseline or 108-month follow-up information. Study cohort characteristics are summarized in Supplemental Table 3.

The models were also evaluated on an external testing group from the MOST database, which consisted of a 270 case–control pairs of subjects with and without TKR performed between the baseline and 84-month follow-up periods. Case subjects and control subjects were matched using baseline age, sex, ethnicity, and BMI with identical inclusion and exclusion criteria as used for selection of the case–control pairs for the training and validation group. Study cohort characteristics are summarized in Supplemental Table 4. The MRI examinations for all subjects consisted of coronal short-tau inversion recovery (COR STIR) and sagittal fat-suppressed intermediate-weighted turbo spin-echo (SAG FS-IW-TSE) sequences performed on a 1.0T dedicated extremity scanner (ONI MSK Extreme; GE Healthcare, Waukesha, WI) with the imaging parameters shown in Supplementary Table 2.

### Analysis of saliency maps

Saliency maps were created for the DESS model that showed the regions of discriminative high activation on the images on which the CNN based its interpretation. The saliency maps were produced by one minus the prediction probability of the DESS model with blocking 32 × 32 × 16 regions sequentially with stride of 8, 8, 4 in the 3 directions. The saliency maps were created for 50 randomly selected subjects and were overlaid on their corresponding DESS reformatted images in axial, coronal, and sagittal planes.

The axial, coronal, and sagittal overlaid saliency maps and DESS images for all image slices through the knee were reviewed by a fellowship trained musculoskeletal radiologist who was blinded to all subject information. The radiologist graded the presence and absence of regions of discriminative high activation in different anatomic locations of the knee including the central bone-cartilage interface and peripheral bone-cartilage interface of the medial and lateral femoral condyles and tibia plateau, medial and lateral meniscus, infrapatellar fat pad, intercondylar notch, and fluid filled joint space.

### Statistical analysis

Statistical analysis was performed using R Project for Statistical Computing Software (R version 3.6.0, R-Project.org). Statistical significance was defined as a *p* value less than 0.05. The Holm method was used to adjust *p* values to account for multiple comparisons^23^.

Model evaluation was performed using sevenfold nested cross-validation on the testing and validation group with additional evaluation performed on an internal hold-out testing group from the OAI database and an external testing group from the MOST database. Models were evaluated for all knees combined and for knees of each individual Kellgren-Laurence (KL) grade for the internal hold-out testing group in the OAI database and for all knees combined for the external testing group in the MOST database. Receiver operator characteristic analysis with areas under the curve (AUC) and area under the precision-recall curve (AUPRC) was used to evaluate the diagnostic performance of the different models developed to predict TKR. 95% confidence intervals for AUCs and AUPRCs were calculated across 5000 bootstrap samples. The Youden index was used to determine optimal model sensitivity and specificity^24^. Statistical significance of improvements in diagnostic performance of the different models was analyzed by comparing AUC differences between models using the Delong test^25^.

Univariate and multi-variate conditional logistic regression models were used to determine the ability of different variables to predict TKR including the outcome prediction of the MRI ensemble model, outcome prediction of the radiograph model, and individual clinical and MRI risk factors for all knees combined and for knees with each individual KL grade. The risk values predicted by the models were normalized to zero mean and unit variance to ensure that differences in mean magnitude prediction are not leading to any discrepancies in odds ratio computation. Clinical risk factors included baseline BMI, WOMAC and contralateral WOMAC scores, and KOOS QoL scores, while MRI risk factors included baseline semi-quantitative MOAKS scores for the severity of cartilage loss and bone marrow edema lesions, which were available in a subset of 270 case–control pairs. The number of articular surfaces with cartilage loss and bone marrow edema lesions was used as MRI risk factors as these variables were shown to provide superior diagnostic performance for predicting incident knee OA than the total MOAK scores for cartilage loss and bone marrow edema lesions^26^. Crude odds ratio (OR) was used to assess the effect of a given variable when it was used as the only predictor, while adjusted OR was used to assess the effect of the given variable adjusted for the effects of all other variables included in the model. Wald test was used to assess the significance of individual variables.

The frequency of regions of discriminative high activation on the saliency maps at each anatomic location of the knee were calculated for case and control subjects. Fisher’s exact test was used to compare differences in the proportions of locations of discriminative high activation in different anatomic locations of the knee between subjects with and without TKR.

## Results

Table 1 shows the AUCs and AUPRCs of the models developed to predict TKR evaluated using sevenfold nested cross-validation on the testing and validation group. All DL models using baseline MRI and radiographs had significantly higher (*p* < 0.001) AUCs for predicting TKR when compared to a traditional machine learning model using clinical risk factors. The MRI and radiograph ensemble model had the highest overall diagnostic performance with an AUC of 0.90 (80% sensitivity and 85% specificity), which was similar (*p* = 0.12) to the AUC of the MRI ensemble model and significantly higher (*p* < 0.05) than the AUCs of the radiograph, IW-TSE, FS-IW-TSE, DESS, and multi-input MRI models. The MRI ensemble model had the second highest overall diagnostic performance with an AUC of 0.89 (79% sensitivity and 86% specificity), which was marginally significantly higher (*p* = 0.06) than the AUC of the DESS model and significantly higher (*p* < 0.05) than the AUCs of the radiograph, IW-TSE, FS-IW-TSE, and multi-input MRI models. The DESS model had the highest diagnostic performance of all individual MRI models with an AUC of 0.88 (82% sensitivity and 81% specificity), which was similar (*p* = 0.24) to the AUC of the IW-TSE model and significantly higher (*p* < 0.05) than the AUCs of the IW-FSE and multi-input MRI models.

**Table 1:** Receiver operator characteristic analysis with areas under the curve (AUC) and area under the precision-recall curve (AUPRC) evaluating the diagnostic performance of the models to predict total knee replacement (TKR) using sevenfold nested cross-validation on the training a validation group in the OAI database.

Model	AUC (95% CI)	*p* value	AUPRC (95% CI)	Sensitivity (%) (95% CI)	Specificity (%) (95% CI)
MLP model					
Traditional	0.77 (0.74, 0.81)	Reference	0.76 (0.71, 0.81)	73 (68, 77)	73 (68, 78)
CNN models					
DESS	0.88 (0.86, 0.91)	< 0.001	0.87 (0.83, 0.91)	82 (78, 86)	81 (77, 85)
FS-IW-TSE	0.86 (0.84, 0.89)	< 0.001	0.87 (0.84, 0.90)	77 (73,82)	84 (80, 87)
Multi-input MRI	0.85 (0.82, 0.88)	< 0.001	0.85 (0.81, 0.89)	79 (75, 83)	79 (74, 83)
IW-TSE	0.87 (0.84, 0.90)	< 0.001	0.87 (0.84, 0.90)	82 (78, 86)	78 (73, 82)
Radiograph	0.87 (0.84, 0.89)	< 0.001	0.87 (0.84, 0.90)	81 (76, 85)	80 (76, 84)
Ensemble models					
MRI	0.89 (0.87, 0.91)	< 0.001	0.89 (0.87, 0.91)	79 (75, 83)	86 (82 ,89)
MRI and radiograph	0.90 (0.87, 0.92)	< 0.001	0.90 (0.87, 0.93)	80 (76, 84)	85 (81, 88)

*MLP* Multi-layer perceptron, *3D* Three-dimensional, *CNN* Convolutional neural network, *CI* Confidence interval, *DESS* Sagittal fat-suppressed three-dimensional dual-echo in steady state, *FS-IW-TSE* Sagittal fat-suppressed intermediate-weighted turbo spin-echo, *IW-TSE* Coronal intermediate-weighted turbo spin-echo.

Table 2 shows the crude and adjusted ORs of the models developed to predict TKR for all knees combined, while Supplemental Table 5 shows the adjusted ORs of the models for knees with each individual KL grade. The outcome prediction of the MRI ensemble model was the variable that had the highest predictive performance in the univariate and multi-variate models with a crude OR and adjusted OR of 17.64 and 10.54, respectively for all knees combined and an adjusted OR of 7.55, 5.84, 3.33, and 3.19 for knees with KL grades of 0, 1, 2, and 3, respectively. For all knees combined, the outcome prediction of the MRI ensemble model, outcome prediction of the radiograph model, BMI, WOMAC score, KOOS QoL score, cartilage MOAK score, and bone marrow edema lesion MOAK score were significant predictors (*p* < 0.05) of TKR in the univariate model. However, the outcome prediction of the MRI ensemble model and cartilage MOAK score were the only variables that remained statistically significant predictors (*p* < 0.05) in the multi-variable model.

**Table 2:** Crude odds ratio (OR) and adjusted OR indicating the ability of different variables to predict total knee replacement (TKR) in univariate and multi-variate conditional logistic regression models for all knees combined.

Parameter	Crude odds ratio	Adjusted odds ratio	*p* value^+^
MRI ensemble	18.24 (9.1, 36.7)	10.81 (4.89, 23.91)	< 0.001
Radiograph	5.56 (3.95, 7.83)	1.45 (0.91, 2.31)	0.119
BMI	1.69 (1.32, 2.16)	1.4 (0.94, 2.09)	0.098
WOMAC	1.32 (1.24, 1.41)	1.02 (0.89, 1.17)	0.776
WOMAC contralateral	1.12 (1.06, 1.18)	1.02 (0.91, 1.14)	0.722
KOOS QoL	0.96 (0.95, 0.96)	0.99 (0.97, 1.01)	0.195
Cartilage subregions	1.48 (1.29, 1.69)	1.06 (0.82, 1.37)*	0.667
BML subregions	1.4 (1.22, 1.61)	1.01 (0.79, 1.3)*	0.909

Data in parenthesis are 95% confidence intervals. Analysis of cartilage and BML subregions was performed on a subset of the study cohort due to missing semi-quantitative MOAKS.

*BMI* Body mass index, *WOMAC* Western Ontario and McMaster Universities Osteoarthritis Index, *KOOS QoL* Quality of life from knee injury and osteoarthritis outcome score, *BML* Bone marrow lesions.

*Adjusted odds ratio from multivariable analysis uses clinical risk factors and image readings from 270 case–control patients.

^+^Wald test was used to assess the significance levels of individual risk factors.

Tables 3 and 4 shows the AUCs and AUPRCs of the models developed to predict TKR evaluated using the internal hold-out testing group in the OAI database and the external testing group in the MOST database, respectively for all knees combined. All models showed a small decrease in diagnostic performance for the internal testing group with AUCs between 0.84 and 0.89 compared to AUCs between 0.85 and 0.90 for the sevenfold nested cross-validation. All models showed a larger decrease in diagnostic performance for the external testing group with AUCs between 0.55 and 0.71. The DESS, FS-IW-TSE, and MRI ensemble models had the highest AUCs between 0.67 and 0.71 for evaluating the sagittal fat suppressed intermediate-weighted TSE images in the MOST database, while the FS-IW-TSE and MRI ensemble models had the highest AUCs of 0.69 and 0.66, respectively for evaluating the coronal STIR images.

**Table 3:** Receiver operator characteristic analysis with areas under the curve (AUC) and area under the precision-recall curve (AUPRC) evaluating the diagnostic performance of the models to predict total knee replacement (TKR) using the internal hold-out testing group in the OAI database for all knees combined.

Model	AUC	AUPRC	Sensitivity (%)	Specificity (%)
CNN models				
DESS	0.84	0.02	69	80
FS-IW-TSE	0.84	0.02	66	82
Multi-input MRI	0.85	0.02	84	78
IW-TSE	0.87	0.04	66	82
Radiograph	0.88	0.03	81	85
Ensemble models				
MRI	0.87	0.02	69	83
MRI and radiograph	0.89	0.03	69	84

*3D* Three-dimensional, *CNN* Convolutional neural network, *CI* Confidence interval, *DESS* Sagittal fat-suppressed three-dimensional dual-echo in steady state, *FS-IW-TSE* Sagittal fat-suppressed intermediate-weighted turbo spin-echo, *IW-TSE* Coronal intermediate-weighted turbo spin-echo.

**Table 4:** Receiver operator characteristic analysis with areas under the curve (AUC) and area under the precision-recall curve (AUPRC) evaluating the diagnostic performance of the models to predict total knee replacement (TKR) using the external testing group in the MOST database for all knees combined.

Model	COR STIR AUC	COR STIR AUPRC	SAG FS-IW-TSE AUC	SAG FS-IW-TSE AUPRC	Radiograph AUC	Radiograph AUPRC
Radiograph					0.88	0.85
3D CNN models						
DESS	0.58	0.56	0.71	0.69		
FS-IW-TSE	0.69	0.69	0.67	0.66		
IW-TSE	0.55	0.55	0.56	0.55		
Ensemble models						
MRI	0.66	0.65	0.71	0.69		

*3D* Three-dimensional, *CNN* Convolutional neural network, *CI* Confidence interval, *DESS* Sagittal fat-suppressed three-dimensional dual-echo in steady state, *FS-IW-TSE* Sagittal fat-suppressed intermediate-weighted turbo spin-echo, *IW-TSE* Coronal intermediate-weighted turbo spin-echo, *COR STIR* Coronal short tau inversion recovery, *SAG FS-IW-TSE* Sagittal fat-suppressed intermediate-weighted turbo spin-echo.

Table 5 shows the AUCs of the models developed to predict TKR for knees of each individual KL grade evaluated using the internal hold-out testing group in the OAI database. The MRI ensemble model had the highest diagnostic performance with AUCs of 0.66, 0.70, 0.78, 0.74, and 0.87 for predicting TKR in knees with KL grades of 0, 1, 2, 3, and 4, respectively. For all models, the diagnostic performance was highest for knees with a KL grade of 4 and lowest for knees with a KL grade of 0.

**Table 5:** Receiver operator characteristic analysis with areas under the curve (AUC) evaluating the diagnostic performance of the models to predict total knee replacement (TKR) using the internal hold-out testing group in the OAI database for knees with each individual Kellgren-Laurence (KL) grade.

Model	KL grade				
	0	1	2	3	4
Radiograph	0.59	0.63	0.73	0.67	0.77
3D CNN models					
DESS	0.64	0.71	0.76	0.70	0.74
FS-IW-TSE	0.63	0.68	0.71	0.71	0.87
IW-TSE	0.66	0.67	0.76	0.73	0.78
Ensemble models					
MRI	0.66	0.70	0.78	0.74	0.87

*3D* Three-dimensional, *CNN* Convolutional neural network, *CI* Confidence interval, *DESS* Sagittal fat-suppressed three-dimensional dual-echo in steady state, *FS-IW-TSE* Sagittal fat-suppressed intermediate-weighted turbo spin-echo, *IW-TSE* Coronal intermediate-weighted turbo spin-echo.

**Figure 3:**
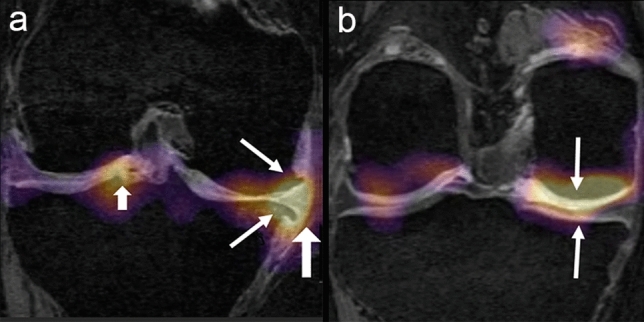
(**a**) Baseline coronal DESS reformatted image of a case subject (WOMAC pain score—9, stiffness score—39.3, disability score—5) with TKR shows regions of discriminative high activation along the peripheral bone-cartilage interface of the medial femoral condyle and medial tibia plateau in areas of osteophyte formation (thin arrows), within the extruded medial meniscus (thick long arrow), and within the lateral tibial spine and intercondylar notch (thick short arrow). (**b**) Baseline coronal DESS reformatted image of a control subject (WOMAC pain score—0, stiffness score—0, disability score—0) without TKR shows regions of discriminative high activation along the central bone-cartilage interface of the medial femoral condyle and medial tibia plateau in areas of healthy appearing cartilage (arrows).

**Figure 4:**
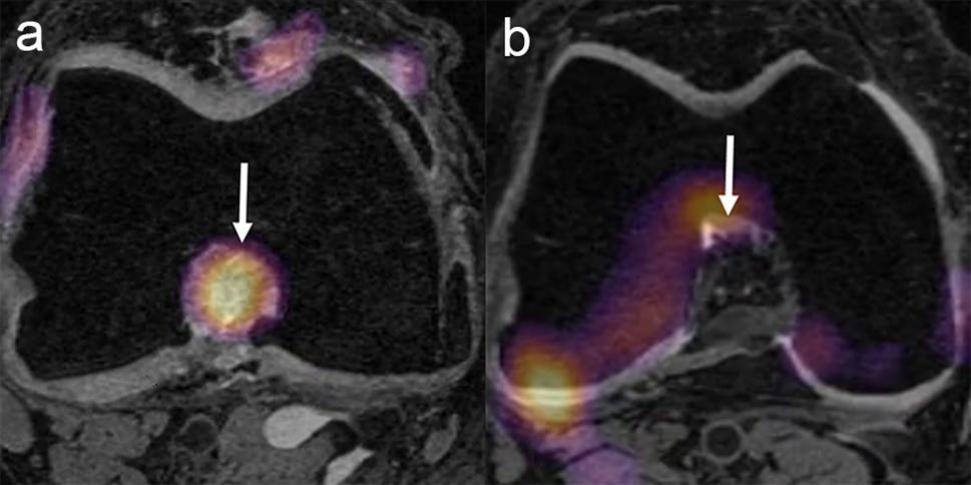
(**a**) Baseline axial DESS reformatted image of a case subject (WOMAC pain score—4, stiffness score—32, disability score—1) with TKR shows a region of discriminative high activation within the intercondylar notch (arrow). (**b**) Baseline axial DESS reformatted image of a control subject (WOMAC pain score—0, stiffness score—0, disability score—0) without TKR does not show a region of discriminative high activation within the intercondylar notch (arrow).

Table 6 shows the frequency of regions of discriminative high activation on the saliency maps at each anatomic location of the knee for case and control subjects. Significantly higher proportion (*p* < 0.05) of case subjects with TKR had regions of high activation on the peripheral bone-cartilage interfaces of the medial and lateral femoral condyles and tibia plateau, intercondylar notch, and medial meniscus compared to subjects without TKR. Significantly higher proportion (*p* < 0.05) of control subjects without TKR had regions of high activation on the central bone-cartilage interfaces of the femoral condyles and tibia plateau compared to subjects with TKR (Figs. 3, 4).

**Table 6:** Frequency of regions of discriminative high activation on the saliency maps at anatomic location of the knee for case subjects with total knee replacement (TKR) and control subjects without TKR computed using the DESS model.

Location	Frequency (%)		*p* value
	Cases	Controls	
Bone-cartilage interface central			
Medial femoral condyle	64.29	100	0.001
Lateral femoral condyle	57.14	100	< 0.001
Medial tibia plateau	64.29	95.45	0.01
Lateral tibia plateau	57.14	95.45	0.003
Bone-cartilage interface peripheral			
Medial femoral condyle	92.86	18.18	< 0.001
Lateral femoral condyle	82.14	22.83	< 0.001
Medial tibia plateau	82.14	23.81	< 0.001
Lateral tibia plateau	71.43	18.18	< 0.001
Medial meniscus	46.43	9.09	0.005
Lateral meniscus	17.86	4.55	0.21
Fat pad	3.57	0.00	0.99
Intercondylar notch	75	4.55	< 0.001
Fluid filled joint space	10.71	4.55	0.62

## Discussion

Our study showed that DL models using baseline MRI had significantly higher (*p* < 0.05) diagnostic performance for predicting the progression of TKR over a 108-month follow-up period when compared to a traditional model using baseline clinical risk factors and a DL model using baseline radiographs. Furthermore, the outcome prediction of the MRI ensemble model in the univariate and multi-variate analysis had much higher crude and adjusted ORs than the outcome prediction of the radiograph model and clinical and MRI risk factors and was one of few variables that was a significant predictor (*p* < 0.05) of TKR when accounting for the effects of all other variables in the multivariate analysis. Thus, our findings indicate that DL analysis of the ensemble of baseline IW-TSE, FS-IW-TSE, and DESS images provides the best independent prognostic information regarding the likelihood of TKR. This emphasizes the need to incorporate DL analysis of baseline MRI into knee OA risk assessment models to maximize diagnostic performance, especially when the imaging modality is available for use.

Our study confirmed the findings of a previous study by Tolpadi et al., which also showed a significantly higher (*p* < 0.05) diagnostic performance for a DL model using baseline DESS images for predicting TKR over a 60-month follow-up period in 4,796 subjects in the OAI when compared to a DL model using baseline radiographs. In this study, a traditional model using clinical risk factors, DL model using radiographs, and DL model using DESS images had AUCs of 0.87, 0.85, and 0.89, respectively for predicting TKR, which were similar to the AUCs in our study of 0.77, 0.86, and 0.88, respectively^27^. Our study further investigated the diagnostic performance of DL models that used different MRI tissue contrasts and found that the DESS model had higher AUC than the IW-TSE and FS-IW-TSE models. The improved diagnostic performance of the DESS model is likely due to the fact that the CNN analyzed higher resolution, 3D volumetric images with a larger number of slices that provided more image data for model training. In addition, DESS has been shown to have high diagnostic performance for detecting various structural features of knee joint degeneration on MRI including cartilage defects, osteophytes, joint effusion and synovitis, ligament and tendon tears, and bone marrow edema lesions^28^.

Our study was the first to investigate DL models that used different MRI tissue contrasts and different imaging modalities together to predict TKR. The MRI ensemble model, which averaged the outcome predictions of the IW-TSE, FS-IW-TSE, and DESS models, and MRI and radiograph ensemble models, which averaged the outcome predictions of the MRI ensemble and radiograph models, had the highest diagnostic performance for predicting TKR. Previous studies have also shown that ensemble models outperform the best individual model inside the ensemble, provided that the individual models are uncorrelated^29,30^. Our study also investigated a multi-input MRI model that analyzed the IW-FSE and DESS images together in the same CNN architecture. However, the multi-input MRI model had lower diagnostic performance than the DESS and MRI ensemble models, which was likely the result of the increase in the number of model parameters, or the inadequate concatenation fusion strategy used in the model architecture. Future work is needed to investigate alternative approaches for DL analysis of different MRI tissue contrasts and different imaging modalities in the same CNN architecture. For example, knowledge distillation and attention-based fusion approaches have been shown to be superior to ensemble methods in DL prediction models combining information from multi-view images^31,32^.

Our study showed a decrease in diagnostic performance when the models were evaluated using an external testing group in the MOST database. This highlights the challenges for widespread application of DL models analyzing baseline MRI, which may be influenced by variability in imaging protocols and scanner types across institutions. However, our results were encouraging as the DESS, FS-IW-TSE, and multi-modality MRI models had moderately high AUCs for predicting TKR for subjects in the MOST database, which could be considered a “worse-case scenario” external testing group with MRI sequences acquired on 1.0T extremity scanners with lower image quality than most clinical MRI protocols performed on 1.5T and 3.0T whole-body scanners. Nevertheless, future research efforts are needed to develop better CNN model architectures that are less sensitive to image fluctuations and new methods of model training using image datasets with more heterogeneous tissue contrasts and image quality to optimize model performance and improve model generalizability.

Our study found differences in the locations of regions of high discriminatory activation on the saliency maps between case and control subjects. Regions of high activation were more common in case subjects with TKR on the peripheral bone-cartilage interfaces, intercondylar notch, and medial meniscus. These likely reflected areas of osteophyte formation, intercondylar synovitis, and meniscal tear and extrusion, which have been previously shown to be risk factors for TKR^9–13,33^. Our results are different from the results of other studies that have used DL models to predict knee pain progression and future TKR. Chang et al. identified regions of high activation in areas of joint effusion in 89% of subjects with knee pain progression^34^. Tolpadi et al. found that the risk of TKR decreased when regions of high activation were present on the bone and cartilage, meniscus, and anterior cruciate ligament and increased when regions of high activation were present on the medial patella retinaculum, gastrocnemius tendon, and plantaris muscle^27^. Differences in our findings and the findings of previous studies may be due multiple factors including differences in CNN architectures, outcome measures for knee OA progression, and methods for saliency map reconstruction and interpretation.

Our DL models could have important future impact in clinical practice and clinical drug trials. The models could improve clinical outcomes and reduce symptoms by identifying patients at high risk for knee OA progression early enough to provide a window of opportunity for disease modification. The models could also provide a paradigm shifting approach that would reduce the size, duration, and costs of future clinical drug trials through exclusive selection of subjects at high risk for knee OA progression, thereby expediting the development of new OA disease modifying therapies. The models could also provide a paradigm shifting approach that would reduce the size, duration, and costs of future clinical drug trials through exclusive selection of subjects at high risk for knee OA progression, thereby expediting the development of new OA disease modifying therapies. Semi-quantitative measures of structural features on MRI have also been shown to be associated with knee OA progression^8–13^. However, obtaining semi-quantitative parameters is time-consuming and reader-dependent, which would make it difficult to incorporate them into widespread, cost-effective OA risk assessment models that could be used in all patients evaluated with MRI in clinical practice. Furthermore, our study has shown that DL models have higher diagnostic performance for predicting future TKR using baseline MRI than semi-quantitative MOAK scores for cartilage loss and bone marrow edema lesions.

Our study has several limitations. Our study defined case subjects as individuals who underwent a TKR over the 108-month follow-up period. Thus, the outcome measure was a binary variable that did not take into account the time after baseline the TKR was performed. Furthermore, the decision to undergo a TKR can be influenced by multiple factors other than the degree of structural joint degeneration on imaging studies such as pain severity, comorbidities, and healthcare access^35^. Our models were also trained and evaluated using the OAI and MOST databases that were primarily composed of older, overweight, Caucasian subjects. Thus, model generalizability to more age, BMI, race, and ethnic diverse populations needs to be further investigated. In addition, our traditional machine learning model used only a limited number of clinical risk factors although all variables were documented in previous studies to be strongly associated with the progression of knee pain and future TKR^21,22^. Finally, our DL models could provide no mechanistic information regarding the factors responsible for progression to TKR.

In conclusion, our study showed that DL models using baseline MRI had significantly higher (*p* < 0.001) diagnostic performance for predicting TKR over a 108-month follow-up period when compared to a traditional model using baseline clinical risk factors and a DL model using baseline radiographs. Furthermore, DL models ensembling different MRI tissue contrasts and different imaging modalities achieved significantly higher (*p* < 0.05) diagnostic performance than DL models that used a single MRI tissue contrast or single imaging modality. However, our study also showed a decrease in diagnostic performance of the models when evaluated on the external testing group in the MOST database, which demonstrates the influence of variables such as subject cohort characteristics and MRI protocols on model performance. Additional work is needed to develop new approaches to combine different image datasets in the same CNN architecture to maximize model performance and to investigate new methods to improve model generalizability to more diverse subject populations and image datasets with more heterogeneous tissue contrasts and image quality.

## Supplementary Information

Supplementary Information.

## Data Availability

The datasets analyzed during the current study are available in the Osteoarthritis Initiative and Multicenter Osteoarthritis Study repositories: https://nda.nih.gov/oai/, https://most.ucsf.edu/multicenter-osteoarthritis-study-most-public-data-sharing. The github repo for our model development and analysis can be found in https://github.com/denizlab/OAI-MRI-TKR.
