# Supplementary material for: Prediction of total knee replacement using deep learning analysis of knee MRI
Source: Sci Rep. 2023 Apr 28;13:6922. doi: 10.1038/s41598-023-33934-1 (PMC10147603; doi:10.1038/s41598-023-33934-1)
Supplement: Supplementary file 1 — Supplementary Information. [file 41598_2023_33934_MOESM1_ESM.docx]

**SUPPLEMENTARY INFORMATION**

## **MRI Total Knee Replacement Risk Assessment Models**

All CNN architectures used conventional residual blocks with extension to 3D. Starting with the input image, the first 3D convolutional layer was followed by a ReLU activation function and a batch normalization layer. The output of the batch normalization layer was fed into a second 3D convolutional layer. The output of the second 3D convolutional layer was added to the input to complete the residual block. Each convolutional layer had a kernel size of 3 and a stride of 1 in all dimensions.

The first two convolutional layers and a max pooling layer were used to bring the spatial volume of the feature maps into a similar range. The first convolutional kernel for the IW-TSE and FS-IW-TSE models was with size 7x7x3 and strides 2x2x2 followed by a max-pooling layer over a 3x3x3 window with stride 2x2x1 and the second convolution kernel was with size 7x7x3 and stride 2x2x1. The first convolutional kernel for the DESS model was of size 7x7x7 and strides 2x2x2 followed by a max-pooling layer over a 3x3x3 window with stride 2x2x2 and the second convolution kernel was with size 3x3x3 and stride 2x2x1. This was followed by batch normalization and ReLU activation.

In the multi-input MRI model, the aforementioned layers were used to reduce the spatial dimension of the feature maps of the FS-IW-TSE and DESS to the same size. The feature maps were then concatenated together for further processing. The influence of the location of the combination of information in the CNN architecture was investigated (Supplemental Figure 1). Combining the information from FS-IW-TSE and DESS images before the first residual block with addition was found to maximize model performance and reduce computational overhead in comparison to combining information in the later stages of the network. For this reason, this method of image combination was used. After combining the information from both images, feature maps were processed using a single path to obtain the final model prediction.

After these steps, all models used the same 8 residual blocks structure and the last residual block was followed by a global max pooling layer and a fully connected layer of size 256. The signal output was a size one linear layer with sigmoid activation function, which was used to define a loss function that aimed to minimize the error between the ground truth and model output during training. The outcome predictions for the models were a confidence value between 0 and 1 indicating the likelihood for TKR.

**Model Training and Evaluation**

Model training and evaluation was performed using 7-fold nested cross-validation. The 353 case-control pairs were split into 7 parts following the dataset splits from a previous study (22), with the number of pairs used for model training, validation and testing ranging between 47 and 52. The dataset splits were performed randomly using a random data generator in Python (version 2.7, Python Software Foundation, Wilmington, DE). [Link will be provided after peer review] provides .csv files that provides subject IDs for each patent and control who are included seven separate groups for nested cross-validation.

DL models were trained using a binary target TKR outcome variable (1: case subjects who underwent TKR within 108-month follow-up, 0: control subjects). Horizontal flipping and random crop were used for data augmentation. To improve model generalizability, random cropping of input image size to 352x352x36, 384x384x36, and 352x352x144 was implemented for the IW-TSE, FS-IW-TSE, and DESS models, respectively. For the multi-input model, FS-IW-TSE input images were randomly cropped to 384x384x36 and the DESS images were randomly cropped to 384x384x144 so that they could be reduced to the same size for further processing. Models were trained using a 7-fold nested cross-validation. The binary cross-entropy loss was used as the training loss. All the models were trained for 200 epochs. Adam optimizer was used with a learning rate of 2 x 10^-4^ with default running average and eps parameters from [36]. The model with the best validation area under the receiver operating characteristic curve (AUC) was selected as the best model from each fold within the nested cross-validation. Final model performance was the average AUC for each model over the 7-folds.

**Supplemental Figure Legends**

**Supplemental Figure 1:** AUC of the trained DL models in a validation set (a) and a testing set (b). The x-axis represents the location of fusing information from multiple contrasts with the following legend: 1 -before residual blocks (as presented in Figure 2b), 2 - after 1st residual block, 3- after 2nd residual block, 4 - after 3rd residual block, 5 - after 4th residual block, 6- combine at the embedding stage (fully connected layer)


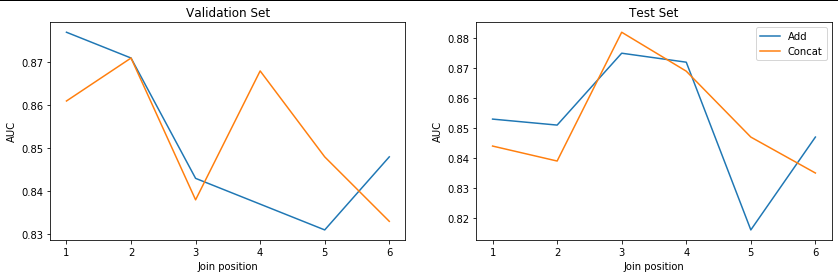


**Supplemental Table 1:** Summary statistics for demographic variables for the matched case-control cohort in the training and validation group in the OAI database.

|  | Men |  |  | Women |  |  |
| --- | --- | --- | --- | --- | --- | --- |
| Parameter | **Patients** | **Control Patients** | ***P* Value** | **Patients** | **Control Patients** | ***P* Value** |
| No. of patients | 138 | 138 |  | 215 | 215 |  |
| Mean age (y) | 64±8 | 64±8 | >0.99 | 63±8 | 63±8 | >0.99 |
| Mean height (m) | 1.76±0.06 | 1.76±0.06 | 0.78 | 1.62±0.06 | 1.62±0.06 | 0.56 |
| Mean weight (kg) | 93.4±14.1 | 92.0±12.7 | 0.38 | 78.5±14.8 | 77.0±13.6 | 0.28 |
| Mean BMI (kg/m^2^) | 29.9±3.8 | 29.4±3.4 | 0.31 | 29.9±5.3 | 29.5±4.7 | 0.41 |
| Ethnicity* |  |  |  |  |  |  |
| White | 126 | 126 |  | 177 | 177 |  |
| African American | 10 | 10 |  | 33 | 33 |  |
| Asian | 0 | 0 |  | 2 | 2 |  |
| Other nonwhite | 2 | 2 |  | 3 | 3 |  |

Note: Mean data are ± standard deviation. P Value compares the differences in mean between case and control groups for each variable. *Number of patients are shown. OAI= Osteoarthritis Initiative, BMI = Body Mass Index.

**Supplemental Table 2:** Imaging parameters of the sagittal fat-suppressed intermediated-weighted turbo spin-echo (FS-IW-TSE), coronal intermediated -weighted turbo spin-echo (IW-TSE), and fat-suppressed three-dimensional dual-echo in steady-state (DESS) sequences performed in the MRI examination of subjects in the OAI database and the coronal short-tau inversion recovery (COR STIR) and sagittal fat-suppressed intermediate-weighted turbo spin-echo (SAG FS-IW-TSE) sequences performed in the MRI examination of subjects in the MOST database.

|  | **TE (ms)** | **TR (ms)** | **TI**  **(ms)** | **FOV (mm)** | **ST (mm)** | **ISR**  **(mm**^2^**)** | **Matrix Size** | **Bandwidth (Hz/pixel)** |
| --- | --- | --- | --- | --- | --- | --- | --- | --- |
| FS-IW-TSE | 30 | 3200 | NA | 160 | 3.0 | 0.36x0.36 | 448x448 | 248 |
| IW-TSE | 29 | 3700 | NA | 140 | 3.0 | 0.36x0.36 | 384x384 | 352 |
| DESS | 4.7 | 16.3 | NA | 140 | 0.7 | 0.36x0.36 | 384x384 | 185 |
| COR STIR | 35 | 4800 | 100 | 140 | 3.0 | 0.55x0.72 | 256x192 | NS |
| SAG FS-IW-TSE | 15 | 7820 | NA | 140 | 3.0 | 0.49x0.72 | 288x192 | NS |

OAI= Osteoarthritis Initiative, MOST= Multicenter Osteoarthritis Study, TE = Echo Time, TR= Repetition Time, TI=Inversion Time, FOV= Field of View, ST= Slice Thickness, ISR= In-Plane Spatial Resolution, NA= Not Applicable, NS= Not Specified.

**Supplemental Table 3**: Summary statistics for demographic variables for the internal hold-out testing group consisting of the remaining subjects in the OAI database that were not involved in model training and validation.

|  | Men |  |  | Women |  |  |
| --- | --- | --- | --- | --- | --- | --- |
| Parameter | **Patients** | **Control Patients** | ***P* Value** | **Patients** | **Control Patients** | ***P* Value** |
| No. of patients | 12 | 1694 |  | 15 | 2340 |  |
| Mean age (y) | 68±9 | 60±10 | <0.01 | 67±7 | 61±9 | <0.01 |
| Mean height (m) | 1.72±0.09 | 1.76±0.06 | 0.05 | 1.59±0.06 | 1.62±0.06 | 0.02 |
| Mean weight (kg) | 86.1±13.7 | 89.5±14.5 | 0.42 | 71.5±13.4 | 74.4±14.7 | 0.44 |
| Mean BMI (kg/m^2^) | 28.9±4.4 | 28.7±4.2 | 0.81 | 28.4±5.5 | 28.2±5.3 | 0.93 |
| Ethnicity* |  |  |  |  |  |  |
| White | 8 | 1396 |  | 12 | 1746 |  |
| African American | 0 | 254 |  | 0 | 524 |  |
| Asian | 1 | 12 |  | 1 | 22 |  |
| Other nonwhite | 12 | 1694 |  | 15 | 2340 |  |

Note: Mean data are ± standard deviation. P Value compares the differences in mean between case and control groups for each variable. *Number of patients are shown. OAI= Osteoarthritis Initiative, BMI = Body Mass Index.

**Supplemental Table 4**: Summary statistics for demographic variables for the matched case-control cohort in the external testing group in the MOST database.

|  | Men |  |  | Women |  |  |
| --- | --- | --- | --- | --- | --- | --- |
| Parameter | **Patients** | **Control Patients** | ***P* Value** | **Patients** | **Control Patients** | ***P* Value** |
| No. of patients | 67 | 67 |  | 203 | 203 |  |
| Mean age (y) | 65±7 | 65±7 | 0.99 | 65±7 | 65±7 | 0.99 |
| Mean height (m) | 1.79±0.06 | 1.78±0.06 | 0.46 | 1.62±0.06 | 1.63±0.05 | 0.41 |
| Mean weight (kg) | 94.8±13.0 | 96.7±18.2 | 0.61 | 81.6±13.6 | 81.6±12.6 | 0.99 |
| Mean BMI (kg/m^2^) | 30.1±4.2 | 30.0±4.1 | 0.98 | 31.1±4.9 | 30.9±4.7 | 0.71 |
| Ethnicity* |  |  |  |  |  |  |
| White | 67 | 67 |  | 188 | 188 |  |
| African American | 0 | 0 |  | 15 | 15 |  |
| Asian | 0 | 0 |  | 0 | 0 |  |
| Other nonwhite | 0 | 0 |  | 0 | 0 |  |

Note: Mean data are ± standard deviation. P Value compares the differences in mean between case and control groups for each variable. *Number of patients are shown. MOST= Multicenter Osteoarthritis Study, BMI = Body Mass Index.

**Supplementary Table 5:** Adjusted odd ratio (OR) for different KL grades indicating the ability of different variables to predict total knee replacement (TKR) in multi-variate logistic regression models. KL grade 4 column is not presented in this table since our study cohort included only 6 TKR control subjects with KL grade 4. This prevented to perform a reasonable statistical comparison.

|  | | **KL Grade** |  | |  |  | |  | |  | |  | |  | |
| --- | --- | --- | --- | --- | --- | --- | --- | --- | --- | --- | --- | --- | --- | --- | --- |
|  | | **0** |  | | **1** |  | | **2** | |  | | **3** | |  | |
| **Parameter** | | **OR** | ***P* Value**^+^ | | **OR** | ***P* Value**^+^ | | **OR** | | ***P* Value**^+^ | | **OR** | | ***P* Value**^+^ | |
| MRI Ensemble | 7.55 (1.80, 36.4) | | | 0.007 | 5.84 (1.59, 25.9) | | 0.012 | 3.33 (1.7, 6.81) | <0.001 | | 3.19 (1.69, 6.3) | | <0.001 | |  |
| Radiograph | 0.25 (0.01, 1.98) | | | 0.247 | 1.33 (0.35, 4.78) | | 0.662 | 1.8 (1.03, 3.18) | 0.041 | | 0.88 (0.42, 1.79) | | 0.727 | |  |
| BMI | 0.90 (0.75, 1.06) | | | 0.244 | 0.90 (0.74, 1.08) | | 0.282 | 0.93 (0.85, 1.01) | 0.103 | | 0.95 (0.88, 1.04) | | 0.291 | |  |
| WOMAC | 0.15 (0.007, 0.6) | | | 0.079 | 0.99 (0.76, 1.28) | | 0.941 | 0.98 (0.87, 1.1) | 0.748 | | 1.08 (0.93, 1.27) | | 0.291 | |  |
| WOMAC Contralateral | 0.97 (0.91, 1.14) | | | 0.898 | 0.91 (0.72, 1.13) | | 0.407 | 1.2 (1.06, 1.37) | 0.006 | | 0.94 (0.82, 1.09) | | 0.422 | |  |
| KOOS QoL | 0.98 (0.93, 1.03) | | | 0.344 | 0.97 (0.93, 1.0) | | 0.06 | 0.98 (0.96, 1.01) | 0.165 | | 0.98 (0.96, 1.01) | | 0.143 | |  |
| Cartilage Subregions* | 1.86 (1.11, 3.75) | | | 0.035 | 1.07 (0.69, 1.64) | | 0.746 | 1.17 (0.89, 1.58) | 0.275 | | 0.74 (0.55, 0.98) | | 0.045 | |  |
| BML Subregions* | 0.44 (0.10, 1.29) | | | 0.177 | 1.04 (0.56, 1.90) | | 0.894 | 0.87 (0.62, 1.21) | 0.407 | | 1.17 (0.88, 1.56) | | 0.276 | |  |

Note: Data in parenthesis are 95% confidence intervals. Analysis of cartilage and BML subregions was performed on a subset of the study cohort due to missing semi-quantitative MOAKS. ^∗^Adjusted odds ratio from multivariable analysis uses clinical risk factors and image readings from 270 case-control patients. ^+^Wald test was used to assess the significance levels of individual risk factors. BMI = body mass index, WOMAC = Western Ontario and McMaster Universities Osteoarthritis Index, KOOS QoL = Quality of Life from Knee Injury and Osteoarthritis Outcome Score, BML = Bone Marrow Lesions.
